# May regional anesthesia be a better choice for the COVID-19 pandemic?

**DOI:** 10.1186/s42077-020-00095-6

**Published:** 2020-10-01

**Authors:** Bahadir Ciftci, Mursel Ekinci, Yunus Oktay Atalay

**Affiliations:** Department of Anesthesiology and Reanimation, Mega Medipol University Hospital, Istanbul Medipol University, School of Medicine, 34040, Bagcilar, Istanbul, Turkey

To the Editor;

The novel coronavirus disease 2019 (COVID-19) is a global pandemic and threat all over the world. The first cases were seen in, Wuhan, China, in December 2019 (Chen et al. 2020a). This novel coronavirus was identified from the throat sample of a patient and was named 2019-nCoV by the WHO. It has spread rapidly, and now, there are more than 2.3 million reported cases and 160,000 deaths worldwide. Anesthesiologists are the frontline warriors both in the intensive care units and operation rooms during this pandemic. There is an important question for us: which anesthesia technique should we choose for these patients? In this report, we would like to share our regional anesthesia experiences in patients under investigation for COVID-19 that underwent surgery.

Written informed consent was obtained from the patients. According to practice recommendations on neuraxial anesthesia and peripheral nerve blocks during the COVID-19 by the American Society of Regional Anesthesia and Pain Medicine (ASRA) and European Society of Regional Anesthesia and Pain Therapy (ESRA) (https://www.asra.com/page/2905/practice-recommendations-on-neuraxial-anesthesia-and-peripheral-nerve-blocks-dur), we preferred regional anesthesia over general anesthesia for these patients to reduce the need for airway manipulation. The blocks were performed in the operating room prepared for just COVID-19 or COVID-19-suspected patients. The drugs and equipment were all prepared in another clean room. Although neuraxial anesthesia and peripheral nerve blocks are not aerosol-generating procedures, we donned personal protective equipment, and the patients wore surgical masks. To minimize the need for conversion to general anesthesia, all the blocks were performed by the most experienced anesthetist.

The first case was a 38-year-old man who underwent wrist fracture operation. He had cough and sub-febrile fever (37.8 °C). After covering ultrasound transducer with plastic covers, ultrasound-guided infraclavicular block was performed.

The second case was a 63-year-old man who underwent proximal femur fracture surgery. He had cough, fever (38.2 °C), and physical examination of bilateral crepitus at the base of the lungs. After ruling out thrombocytopenia, we performed spinal anesthesia at the level of L4–L5 intervertebral space.

The third case was a 25-year-old cesarean section case. Her physical examination and vital signs were normal; however, her husband was COVID-19 positive. Her thrombocyte level was normal, and we performed her spinal anesthesia at the level of L3–L4 intervertebral space. The parturient had hypotension (75/50 mmHg) and recovered with 20 mg ephedrine. Because virus is isolated from cerebrospinal fluid (CSF), we did not allow the CSF to drip freely to reduce contamination in both of spinal anesthesia cases. Because continuous catheter techniques can increase the risk of frequent patient contact, we did not prefer epidural and perineural catheters.

## Discussion

When it is thought that the transmission of this virus is via aerosols, dealing with these patients not only in intensive care units but also in the operating rooms puts the anesthetists in the most risk group about transmission. Therefore, any anesthesia technique that avoids the need for airway manipulations is crucial during this pandemic; in this situation, it is the regional anesthesia techniques such as central neuraxial blocks, peripheral nerve blocks, and interfascial plane blocks (Lie et al. 2020; Altiparmak et al. 2020; Chen et al. 2020b). These techniques are also the cornerstones for any patient suspected to be COVID-19 positive. Regional anesthesia techniques may have an important role during the pandemic. However, possible low platelet counts should be kept in mind in 2019-nCoV like the other viral infections (Altiparmak et al. 2020). In our patients, the platelet counts were in normal ranges. In summary, regional anesthesia techniques may be safer for both anesthesiologists and 2019-nCoV patients, if the required precautions are taken.

## Data Availability

Not applicable.

## Abbreviations

2019-nCoV: Novel coronavirus disease 2019
ASRA: American Society of Regional Anesthesia and Pain Medicine
ESRA: European Society of Regional Anesthesia and Pain Therapy
